# Supplementary material for: Inverted temperature gradients in gold–palladium antenna-reactor nanoparticles
Source: Nat Commun. 2025 Sep 1;16:8168. doi: 10.1038/s41467-025-63327-z (PMC12402298; doi:10.1038/s41467-025-63327-z)
Supplement: Supplementary file 1 — Supplementary Information [file 41467_2025_63327_MOESM1_ESM.pdf]

Supplementary information to "Inverted Temperature Gradients in Gold–Palladium Antenna–Reactor Nanoparticles"

Felix Stete,[1] Shivani Kesarwani,[1] Charlotte Ruhmlieb,[2] Sven H. C. Askes,[3] Florian Schulz,[2] Matias Bargheer*,[1,4] and Holger Lange*[1,5]
[1)] *Institut für Physik & Astronomie, Universität Potsdam, 14476 Potsdam, Germany*
[2)] *Institut für Physikalische Chemie, Universität Hamburg, 20146 Hamburg, Germany*
[3)] *Department of Physics and Astronomy, Vrije Universiteit Amsterdam, De Boelelaan 1081, 1081 HV Amsterdam, Netherlands*
[4)] *Helmholtz Zentrum Berlin, Albert-Einstein-Str. 15, 12489 Berlin, Germany*
[5)] *The Hamburg Centre for Ultrafast Imaging, Universität Hamburg, 22761 Hamburg, Germany*
* *bargheer@uni-potsdam.de, holger.lange@uni-potsdam.de*

Supplementary Note 1, plain gold nanoparticles

Supplementary Figure 1 displays an exemplary TEM image of the employed plain gold nanoparticles. The samples feature a uniform spherical shape and a narrow size distribution as estimated from a statistical TEM analysis.

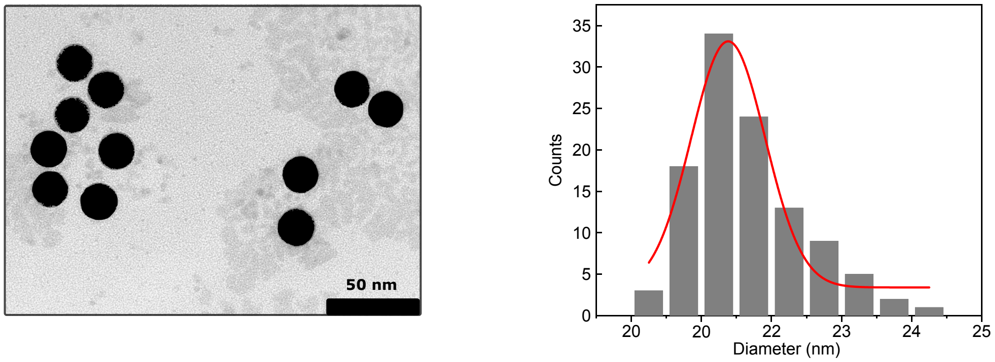

Supplementary Figure 1. **Characterization of pure gold particles.** TEM image of 21 nm diameter gold nanoparticles and size distribution estimated from a broader set of TEM data.

Supplementary Note 2, XRD characterization

To confirm the crystallinity of the gold nanoparticles with palladium satellites, we employed powder X-ray diffraction. Supplementary Figure 2 displays a representative XRD scattering spectrum of the $Pd_{19\%}$ sample. The observed Bragg reflections can be indexed on the basis of the face centered cubic fcc structure of gold. The diffraction peaks at $2\theta=$ 38.2° (1 1 1), 44.4° (2 0 0), 64.5° (2 2 0) and 77.2° (3 1 1) are identical with those reported for the standard gold metal (Au^0) (Joint Committee on Powder Diffraction Standards-JCPDS no. 04-0784, USA).This confirms that the gold nanoparticles are crystalline. The palladium particles are too low in relative mass and the palladium domain sizes are too small to significantly contribute to the scattering spectrum.

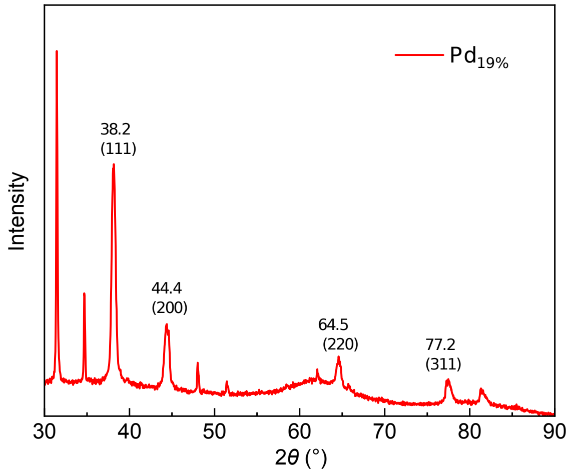

Supplementary Figure 2. **XRD characterization.** XRD spectrum from the $Pd_{19\%}$ bimetallic gold-palladium nanoparticles. The labels correspond to the gold fcc indices.

Supplementary Note 3, EDX spectra

Supplementary Figure 3 displays an exemplary EDX spectrum of the sample $Pd_{19\%}$. The corresponding element analysis is summarized in Supplementary Table I.

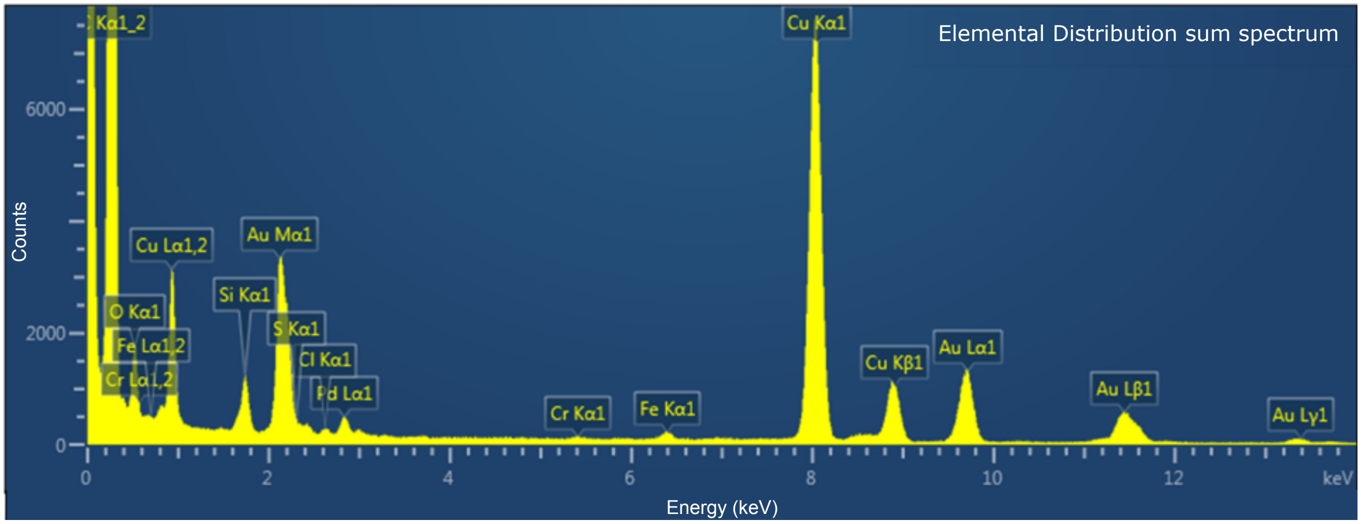

Supplementary Figure 3. **EDX spectra.** EDS measurement of the $Pd_{19\%}$ bimetallic gold-palladium nanoparticles. The peaks are labeled according to the contributing elements.

Element	Line type	k-factor	k-factor type	Absorption correction	Mass %	Mass % sigma	Atom %
Pd	*L*-series	0.654	theoretical	1	10.93	0.52	18.52
Au	*M*-series	0.618	theoretical	1	80.07	0.52	81.48

Supplementary Table I. Elemental analysis of the spectrum diplayed in Supplementary Figure 3.

The atomic mass ratios were used to calculate the volumes for the three-temperature model.

Supplementary Note 4, plain palladium nanoparticles

Supplementary Figure 4 displays exemplary TEM images of the palladium nanoparticles used as reference sample.

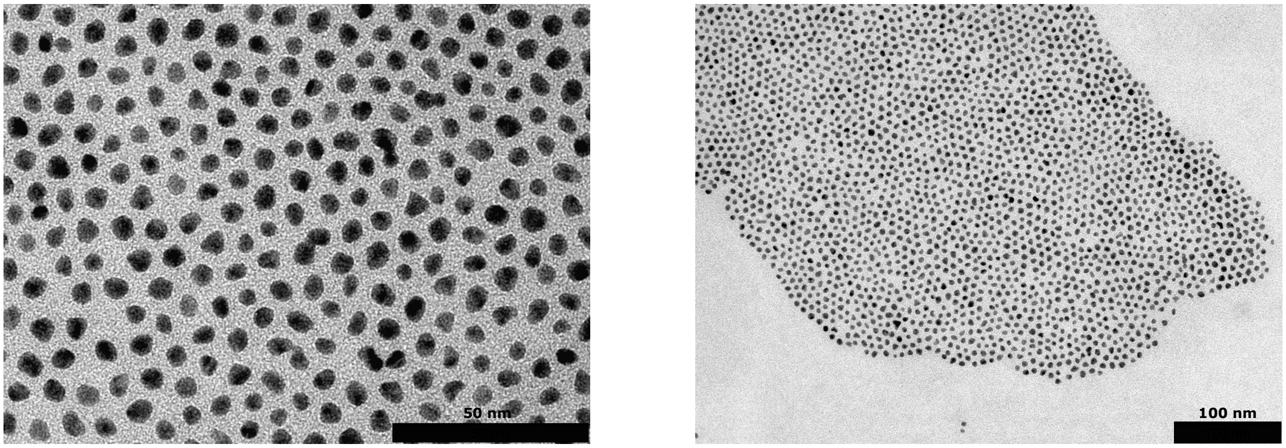

Supplementary Figure 4. **Plain palladium nanoparticles.** TEM images of palladium nanoparticles in two magnifications.

Supplementary Figure 5 displays a stationary absorption spectrum and exemplary transient absorption traces of the plain palladium nanoparticles. In the transient absorption experiment, the palladium nanoparticles show no response in the wavelength range relevant for the discussion of the dynamics in the main text.

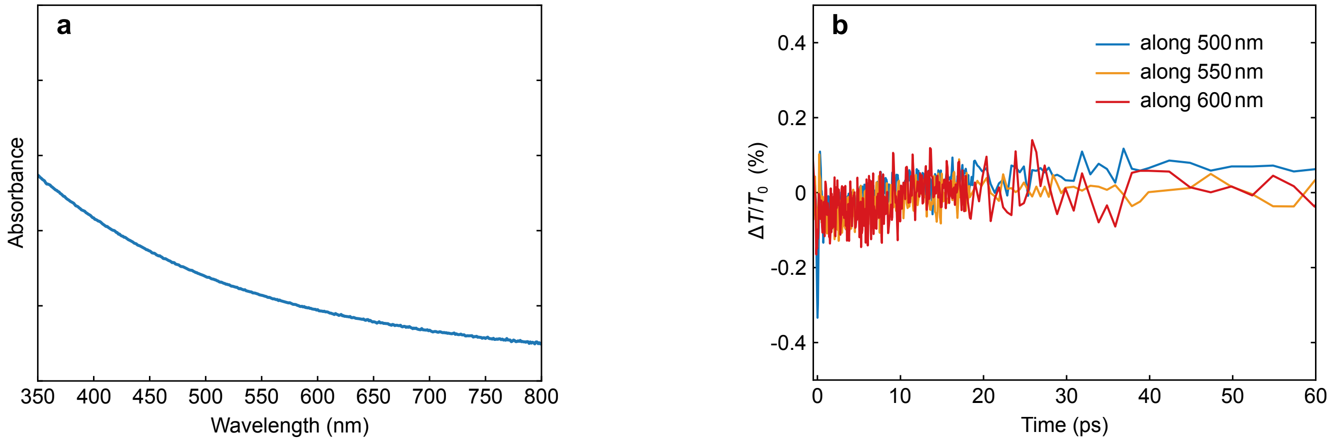

Supplementary Figure 5. **Spectral response of plain palladium nanoparticles.** a) Absorption spectrum of palladium nanoparticles and b) Transient absorption bleach dynamics along selected wavelengths. The nanoparticles were excited at 400 nm.

Supplementary Note 5, exemplary transient spectra of bimetallic gold-palladium nanoparticles

Supplementary Figure 6a displays the full data set of an exemplary transient absorption measurement. The heatmap depicts the changes in transmission of the sample $Pd_{19\%}$ after excitation with a $1.01\,\mathrm{mWcm^{-2}}$ pump pulse. Cuts along the spectrum at a time delay of 0.5 ps, 2 ps and 6 ps are presented in Supplementary Figure 6b. The transients discussed in the main text were extracted as the values at the maxima of each spectral cut.

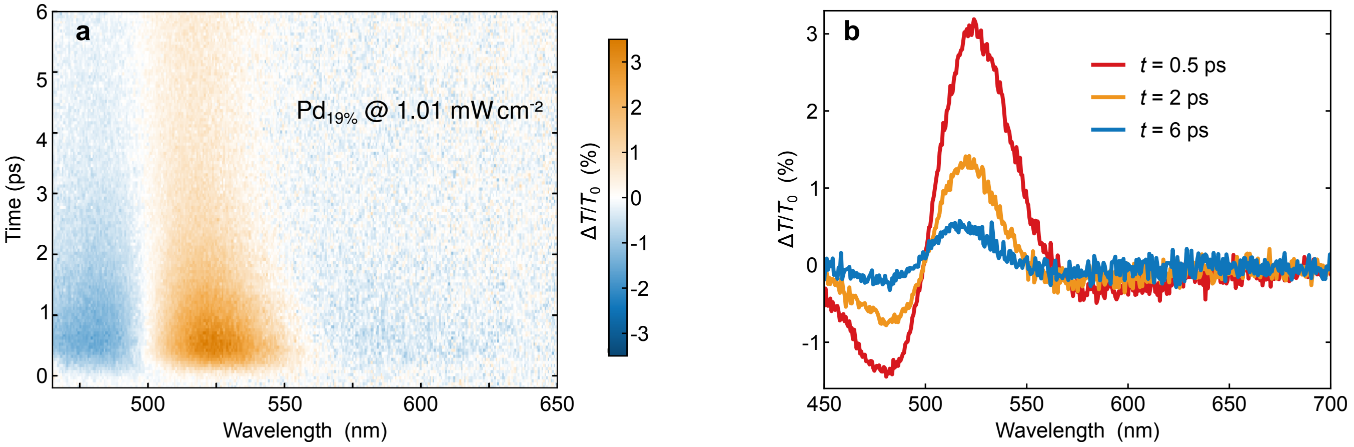

Supplementary Figure 6. **Exemplary transient measurement.** a) Exemplary heatmap of transient absorption experiment of sample $Pd_{19\%}$ pumped with a fluence of $1.01\,\mathrm{mW/cm^2}$. b) Cuts along the spectrum 0.5 ps (red), 2 ps (orange) and 6 ps (blue) after excitation with the pump pulse.

Supplementary Note 6, measured and modelled transients for $Pd_{88\%}$ and $Pd_{144\%}$

Supplementary Figure 7 displays the measured transients and modeled changes in the electron gas of the samples $Pd_{88\%}$ and $Pd_{144\%}$ which complement Figure 3 in the main text.

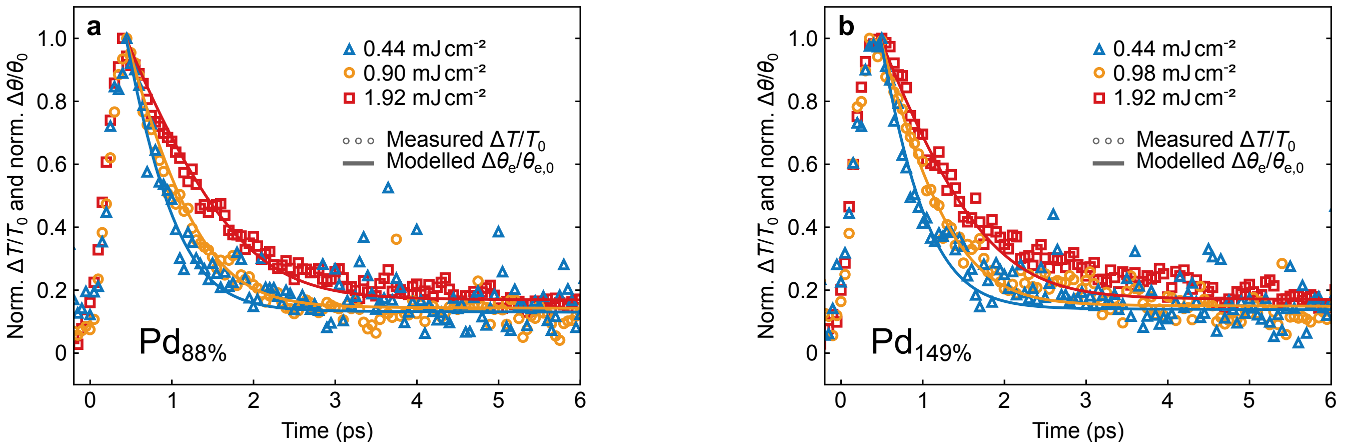

Supplementary Figure 7. **3TM for higher palladium load.** Measured and modelled transients for $Pd_{88\%}$ (a) and $Pd_{144\%}$ (b).

Supplementary Note 7, optical absorption in the gold-palladium nanoparticles

To evaluate the localization of the absorption in $Pd_{19\%}$ nanoparticles, we performed optical simulations as explained in the methods section on electromagnetic simulations. Figure 8 shows the excellent agreement of Comsol and Lumerical simulations.

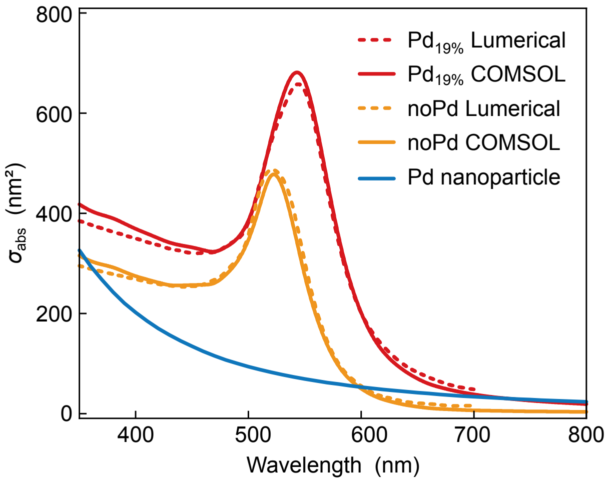

Supplementary Figure 8. **FDTD simulations.** Simulated absorption cross section (σ_{abs}) of $Pd_{19\%}$ nanoparticles. a) Geometry of the $Pd_{19\%}$ nanoparticle in the Lumerical simulation environment. The Au core and Pd satellites have 10.5 nm and 2.5 nm radii, respectively. b) Absorption cross sections of $Pd_{19\%}$ (red), noPd (orange), and a plain palladium nanoparticles (blue) (10.5 nm radius) as retrieved by Lumerical FDTD (dashed) and COMSOL FEM simulations (solid). c) Contributions to the absorption cross section of $Pd_{19\%}$ (red line) in the gold core (orange line) and palladium satellites (blue line).
